# Fresh/High-Zinc Maize: A Promising Solution for Alleviating Zinc Deficiency through Significant Micronutrient Accumulation

**DOI:** 10.3390/foods12142757

**Published:** 2023-07-20

**Authors:** Aldo Rosales, Aide Molina-Macedo, Mayolo Leyva, Félix San Vicente, Natalia Palacios-Rojas

**Affiliations:** International Maize and What Improvement Center (CIMMYT), Texcoco C.P. 56237, Mexico; a.rosales@cgiar.org (A.R.); a.l.molina@cgiar.org (A.M.-M.); m.leyva@cgiar.org (M.L.); f.sanvicente@cgiar.org (F.S.V.)

**Keywords:** green maize, *Zea mays*, biofortification, micronutrients

## Abstract

Zinc deficiency poses a significant health challenge worldwide, particularly in regions where access to and the affordability of dietary diversity are limited. This research article presents a time course analysis of kernel development on the zinc content in maize kernels with different genetic backgrounds, including normal maize, quality protein maize, and high-zinc maize, grown at two locations. Zn concentrations during stage I were high, decreasing between stages II and IV and increasing during stages V to VII. High-zinc kernel genotypes, including those ones with high-quality protein genetic backgrounds, have higher contents of zinc and iron during the milky stage (fresh/green maize). The zinc and iron content in fresh maize differed depending on the genotype. By consuming fresh maize biofortified with zinc, up to 89% and 100% of EAR needs can be fulfilled for pregnant women and children. The results demonstrate that fresh high-zinc maize accumulates a substantial amount of this micronutrient, highlighting its potential as a valuable source for addressing zinc deficiency.

## 1. Introduction

Zinc (Zn) is present in a variety of dietary sources, including red meat, oysters, crabs, lobsters, and other seafood. Legumes, such as chickpeas, lentils, and beans, can also serve as good sources of zinc. However, it is important to note that the zinc content in these foods may vary depending on factors such as food processing and preparation methods. Additionally, the accessibility and affordability of these zinc dietary sources can be limited for certain populations. Factors such as geographic location, economic constraints, and cultural preferences may impact individuals’ ability to access and afford foods rich in zinc [1,2]. Zinc deficiency is a worldwide nutritional disorder affecting one-third of the world’s population, mainly in low- and middle-income countries (LMICs) [1,2,3]. Zn plays an important role in many biological processes and brain functioning and is an essential trace element for the immune systems [4]. The prevalence of inadequate zinc intake in Africa alone was estimated to be 37–62% [5], contributing significantly to poor child growth and immune system weakness [6,7,8]. Zn deficiency is also a major public health problem in vulnerable populations of Latin America and the Caribbean, especially in children under 6 years of age and women of reproductive age [9,10]. Very recently, evidence has shown that Zn supplementation could be effective in combatting COVID-19-related symptoms, inflammation, and neurological damage due to the neuroprotective properties of this trace element [4].

Maize (*Zea mays* L.) is a dietary staple for more than 200 million people, especially in Sub-Saharan Africa, Mesoamerica, and some countries in Latin America. It provides around 15% of the world’s protein and at least 30% of food calories in LMICs [11]. Thus, increasing Zn levels in maize grain could deliver more Zn to people whose diet relies directly or indirectly on maize-derived food and could help mitigate Zn deficiency [3,12]. Although kernel zinc content is affected by genetic and environmental effects like soil, there is high genetic diversity in terms of the zinc content in the kernels (mean 25.50 ± 0.44 mg kg^−1^; range 15.60–48 mg kg^−1^) [13], which has allowed for the development of high-Zn maize varieties that are currently available in different countries, including Guatemala, Colombia, Nicaragua, Honduras, and El Salvador and there are pre-commercial varieties in Haiti, Panamá, México, South Africa, Egypt, and Nigeria [12,14,15].

Zinc in maize kernels is mainly accumulated in the germ and the outer layer [16]; therefore, there will be a higher intake of zinc when the whole kernel is consumed [17]. Regarding iron (Fe), biofortification research has primarily focused on crops that have been shown to be relatively high in iron and demonstrate genetic diversity in terms of Fe content (for example, beans and pearl millet). In contrast to other crops, maize has not been extensively pursued in terms of Fe biofortification because the Fe concentration is approximately 60% lower than that required to have a high nutritional impact, especially due to the presence of Fe bioavailability inhibitors that can be present in processed maize [18,19]. The average content of Fe in maize is about 16 mg kg^−1^; however, a slight increase in Fe in high-zinc maize has been observed, up to 22 mg kg^−1^. This can be attributed to pleiotropic effects or linkage among the genes governing the kernel Zn and Fe uptake and mobilization to kernels [20] and can vary depending on the soil characteristics. Nevertheless, the biofortification of Zn may have an impact on the Fe content in maize.

One common way of maize consumption worldwide is fresh or green maize, where kernels are in the physiological milky stage. It is normally consumed boiled or roasted [21,22], and it is very popular in Latin America, South Asia, China, and several African countries. Sweetcorn, a variant of fresh maize, is the third large vegetable consumed in the USA, and although it is not a major contributor to the American diet, it has recently been the subject of studies toward the development of enhanced mineral sweetcorn [16,23]. Consumers appreciate fresh maize because of its delicate flavor and sweet fragrance. It is also part of traditional festivities, especially in Mesoamerican countries, and it provides economic opportunities for street vendors worldwide. The nutritional value of fresh maize includes the content of amylopectin, sugar, protein, vitamins, cellulose, calcium, iron, and phosphorus [24,25]. When quality protein maize or provitamin-A-enhanced maize is consumed as fresh maize, they also contribute tryptophan, lysine and provitamin A carotenoids [21].

This study aimed to assess the zinc content during different grain filling stages in zinc kernel maize and estimated its potential nutritional impact when consumed fresh.

## 2. Materials and Methods

### 2.1. Plant Materials

Nine tropical experimental maize varieties were used: Six zinc-enriched, three of them also being quality protein maize (meaning, higher tryptophan, and lysine content; NuQMZn), three were only high in Zn (NuMZn), and three were normal (Normal) maize. All the genotypes were grown in 2 replicates at two locations in Mexico, Tlaltizapan (TL), Morelos and Agua Fria (AF), Puebla, during the summer cycle of 2018.

Soil type and properties have been previously characterized, taking four (AF) and six (TL) distal points at 0–30 cm depth in the field trials. Samples were dried and sent for analysis at Fertilab commercial laboratory in Mexico. Both sites have clay loam texture soils with the specific characteristics shown in Table 1.

The time points for grain sampling corresponded with Nielsen et al.’s grain filling stages [26]. The counting of days after pollination (DAP) started from the date when 50% of the plants were flowering (silk started to emerge, and pollen was present). Ear samples were collected at pre-blister (8–12 DAP -I-), blister (12–16 DAP -II-, 80–85% kernel moisture), kernel milk (16–20 DAP -III-, 75–80% kernel moisture), kernel dough (25–29 DAP -IV-, 60–65% kernel moisture), kernel dent (36–40 DAP -V-, 50–55% kernel moisture), physiological maturity (58–62 DAP -VI-, 30–35% kernel moisture), and at harvest maturity (63–67 DAP -VII-, 18–25% kernel moisture).

Two to four ears per replicate per genotype were collected in each stage and used for kernel sampling. Kernels from the middle part of the ears were sampled for micronutrient analysis. All samples, except for kernels from harvest maturity stage, were freeze-dried on dry ice before transferring them to the laboratory. Prior to the chemical analysis, samples were lyophilized for 6 to 9 days at −80 °C using a VirTis Benchtop 2KBTXL freeze-dryer. Dry materials were milled using a Retsch^®^ grinding mill (MM300) with Teflon chambers and zirconium balls. Before the analysis, samples were dried at 70 °C overnight.

### 2.2. Iron and Zinc Concentration in Maize Kernels

Fe and Zn concentrations were determined as described by Palacios-Rojas et al. [27]. Briefly, samples (300 mg) were weighed into 100 mL Pyrex tubes. Digestion was initiated by adding 5 mL of HNO_3_:HClO_4_ mixture (9:1 *v*/*v*). Samples were vortexed and covered with polyethylene wrap and incubated for pre-digestion overnight at room temperature under a fume hood. Digestion was performed by gradually heating the digestion block from 80 to 225 °C for 4 h. After cooling, 10 mL of HNO_3_ was added, and the sample was mixed. The concentrations of iron and zinc are expressed as mg kg^−1^ (DW) and were determined via inductively coupled plasma optical emission spectroscopy (ICP-OES; Optima™ 8300 DV, Perkin Elmer, Waltham, MA, USA). An axial view and the following spectral lines were used: Fe 259.939 nm and Zn 213.57 nm. Flow rates of plasma gas, nebulizer gas, auxiliary gas and peristaltic pump flow rate were fixed at 15.0 L/min, 0.50 L/min, 0.80 L/min, and 1.00 mL/min, respectively. Dried air supplied by an air compressor was used as shear gas for the ICP OES system. A certified material was included in the analyses as a reference (Wheat Flour 1567a, NIST, Gaithersburg, MD, USA).

### 2.3. Estimated Average Requirement

We calculated the percentage of the Estimated Average Requirement (EAR) of Fe and Zn given by fresh maize consumption. EAR is the daily intake value that is estimated to meet the requirement, as defined by the specified indicator or criterion of adequacy, in half of the apparently healthy individuals in a life stage or gender group [28].

### 2.4. Statistical Analysis

All results were statistically analyzed utilizing SigmaPlot ver. 11 (SystatSoftware, Inc., Palo Alto, CA, USA) to identify significant differences between the groups using ANOVA and Tukey’s test (*p* < 0.05).

## 3. Results

### 3.1. Zinc Accumulation during Kernel Development

**Figure 1:**
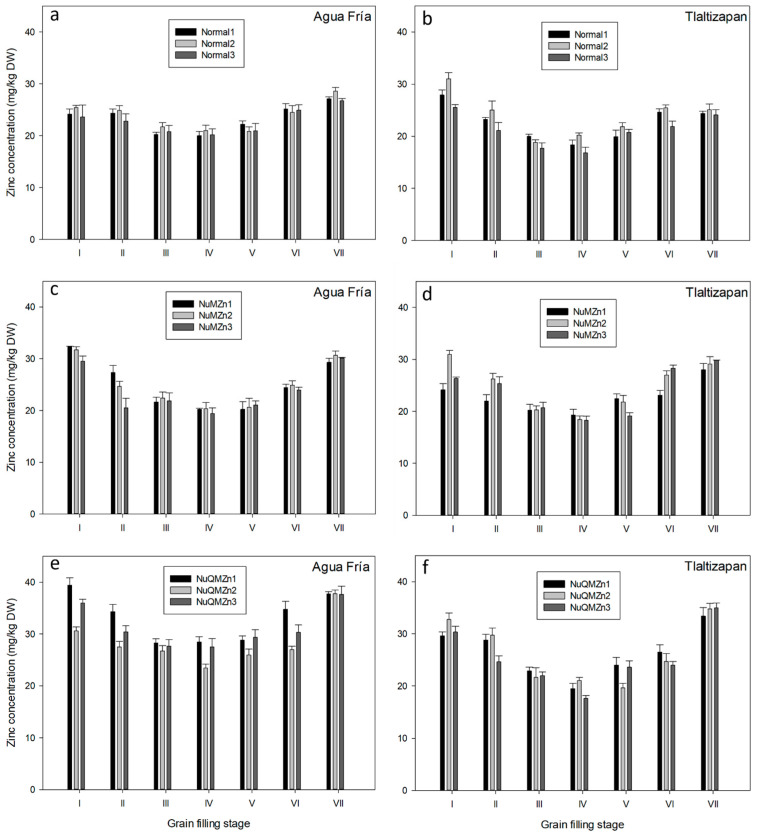
Zinc content during different grain filling stages. Lower case letters indicate Tukey grouping (*p* < 0.05) among types of genotypes (Normal, High Zn, and HighZn + QPM) at different locations (AF and TL). Normal genotypes: graphs (**a**,**b**). High Zn genotypes: graph (**c**,**d**). HighZn + QPM genotypes: graphs (**e**,**f**). The bars indicate the standard deviation of 10 to 12 determinations by genotype type.

A similar trend for Zn accumulation during kernel development was observed for all genotypes and independently of growing location. Zn concentration during stage I was high, decreasing between stages II and IV and increasing during stages V and VII (Figure 1).

### 3.2. Zinc Content at Stage III (Fresh Maize) and Stage VII (Harvest Maturity)

**Figure 2:**
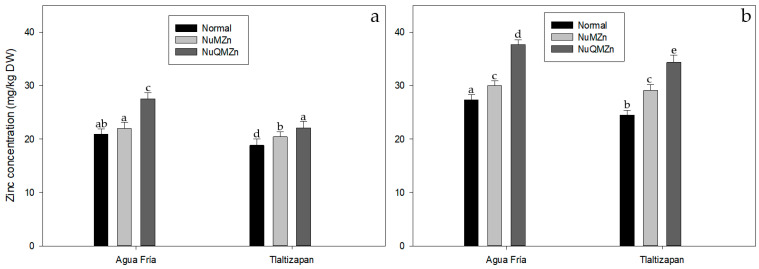
(**a**). Average kernel zinc content at stage III. Letters indicate Tukey grouping (*p* < 0.05) among genotype types at different locations. The bars indicate the standard deviation of 10 to 12 determinations by kind genotype. (**b**). Average kernel zinc content at stage VII. Letters indicate Tukey grouping (*p* < 0.05) among kinds of genotypes at different location. The bars indicate the standard deviation of 10 to 12 determinations by kind genotype.

Zinc concentration during the milky and harvest maturity stage of the kernels varied according to genotype. As expected, the normal genotypes had a lower content compared to NuMZn and NuQMZn (Figure 2a,b). At stage III (milky stage), the kernel Zn concentration for all genotypes was higher in one location compared to the other location. In AF, Normal and NuMZn materials had similar Zn contents, while NuQMZn had a higher Zn content. In TL, Normal maize had the lowest Zn concentration, and NuQMZn had the highest one (Figure 2a).

At stage VII (harvest maturity), Normal and NuQMZn materials growing in AF had higher Zn concentrations compared with the same material growing in TL. For NuMZn materials, there was no significant difference (Figure 2b).

### 3.3. Iron Content in Stage III and VII

Kernel iron content was also measured, and its contribution to the Estimated Average Requirement was calculated (Table A1). During stage III, the NuMZn material had significantly higher Fe content, 14.05 ± 0.71 mg kg^−1^ in AF and 13.96 ± 0.66 mg kg^−1^ in TL, compared with Normal, 13.13 ± 0.69 mg kg^−1^ in AF and 11.75 ± 0.77 mg kg^−1^ in Tl, and NuQMZ, 12.84 ± 0.73 mg kg^−1^ in AF and 12.55 ± 0.98 mg kg^−1^ in TL. In stage VII, NuMZn and NuQMZ had similar Fe concentrations (not significant differences), 20.79 ± 1.07 mg kg^−1^ in AF and 19.74 ± 1 mg kg^−1^ in TL.

### 3.4. Contribution of Fresh High-Zinc Maize to the Estimated Average Requirement (EAR) of Zinc and Iron

The consumption of Normal green maize can provide between 49.48 and 60.8% of the Zn EAR to pre-school children and pregnant women and between 61.85 and 86.86% to children aged 4–13 years, while the consumption of NuMZn fresh maize can contribute between 56.65–62.78% and 70.81–89.68%, respectively. The consumption of NuQMZn had a significantly higher contribution: 60.63–79.04% and 75.79–112.91% for the same groups (Table 2).

In the case of Fe, the contribution to the EAR is between 13.85 and 33.28% for pre-school children and pregnant women and for children aged 4–13 years, and the percentage was significative, 38.84–67.69% (Table A1).

## 4. Discussion

Zn-deficient soils are those that have extractable Zn levels lower than 1.5 mg kg^−1^ [29,30,31]; thus, soils in AF and TL can be classified as Zn-deficient (Table 1). Genotypes growing in AF had a higher Zn content compared with TL, although, in both locations, the Zn concentration in the soil was similar (0.84 mg kg^−1^ in AF and 0.76 mg kg^−1^ in TL). However, total Zn content is not a reliable index to reflect the ability of the soil to supply Zn for plant uptake. A high calcium carbonate (CaCO_3_) content (>20%) and a pH of 7.5–8.1 could cause low Zn availability [29], as could be the case of the soil in TL, where the total carbonate concentration was 28.98%, and the pH was 8.23. The difference in Zn levels between these two locations is in accordance with previous research performed at the same experimental sites [32].

The zinc content profile during the grain filling stage showed almost the same trends for all genotypes, with lower Zn concentration at stage III. These results suggest that dry matter accumulation was faster than Zn accumulation, whereas, from stage IV onwards, both Zn and dry matter accumulated in the kernel at relatively constant proportions [33].

Independent of genotype or location, the general trend in zinc content during grain filling, as reported separately, was a lower content of zinc between stage III (kernel milk) and stage IV (kernel dough). Following stage IV, the zinc content increased until reaching harvest maturity (stage VII). The study conducted by Xue et al. [3] focused on investigating the zinc content in grains during different stages of development after silking, considering one genotype, two cropping seasons, and varying nitrogen regimes. They found that initially, after silking, there was a high zinc content in the grains. However, approximately 15 days after silking, the zinc content started to decrease. Subsequently, there was a slight increase in zinc content after this decrease. The findings also indicated that the zinc content at kernel maturity and the milky stage was similar.

NuMZn and NuQMZn had higher contents of zinc compared to the normal genotype, and this was observed at the milky stage as well as in the mature kernels. The results in mature kernels are like those reported by Mageto et al., 2020 [32], and, as expected, are higher than in normal maize. Although the levels of Zn at the milky stage were about 10–15 mg kg^−1^ less than in mature grains, by consuming fresh biofortified maize, up to 100% of EAR needs can be fulfilled for children. Consuming whole kernels from high-Zn maize by means of alkaline-cooked (nixtamalized) tortillas can contribute up to 76% to the EAR for children 4–6 years old and 89% for women of child-bearing age [17].

Some of the high-Zn maize could provide up to 67.7% of the required amount of EAR iron in children aged 9 to 13 years; however, more information on Fe bioavailability is needed. According to Glahn et al. [34] and Keigler et al. [19], corn germ, as well as pericarp- and endosperm-type characteristics, may have inhibitory effects on Fe bioavailability. On the other hand, some processing methods may increase bioavailability [32].

Given the nutritionally enhanced properties of whole grain [35], its consumption should be further encouraged. Additionally, such benefits are even higher if biofortified maize like high-Zn or provitamin A [16] varieties are consumed. Through this research, it becomes evident that fresh/green high-zinc maize holds promise as a sustainable and accessible solution to combat zinc deficiency in regions heavily reliant on maize consumption. It is important the promotion and integration of fresh/green high-zinc maize into agricultural practices and dietary interventions that encourage as much as possible dietary diversification.

## Figures and Tables

**Table 1:** Soil properties from the field trial sites.

Trait	AF	TL
pH	8.05	8.23
Bulk density (g cm^3^)	1.18	1.07
Organic matter concentration (%)	3.60	2.25
*p*-Olsen (mg kg^−1^)	28.85	25.48
Salinity (dS m^−1^)	0.84	0.98
Total carbonates (%)	7.07	28.98
K (mg kg^−1^)	398.25	414.17
Ca (mg kg^−1^)	6437.25	5155.67
Mg (mg kg^−1^)	251.00	629.17
Na (mg kg^−1^)	35.13	36.27
Fe (mg kg^−1^)	18.00	3.21
Zn (mg kg^−1^)	0.84	0.76
Mn (mg kg^−1^)	2.98	3.97
Cu (mg kg^−1^)	0.98	0.83
B (mg kg^−1^)	0.85	0.71
S (mg kg^−1^)	5.39	13.91
N-NO_3_ (mg kg^−1^)	30.08	13.62

**Table 2:** Estimated Average Requirement (EAR) for children and pregnant women.

Genotype	Zinc in Fresh Corn (mg kg^−1^ DW)	Pre-School Children (1–3 Years Old)	Children (4–8 Years Old)	Children (9–13 Years Old)	Pregnant Women (19–50 Years Old)
% EAR ^a^					
Normal1-AF	20.23	56.63	70.79	80.91	56.63
Normal2-AF	21.72	60.80	76.00	86.86	60.80
Normal3-AF	20.75	58.09	72.61	82.98	58.09
Normal1-TL	19.96	55.90	69.88	79.86	55.90
Normal2-TL	18.86	52.79	65.99	75.42	52.79
Normal3-TL	17.67	49.48	61.85	70.68	49.48
NuMZn1-AF	21.64	60.58	75.73	86.55	60.58
NuMZn2-AF	22.42	62.78	78.47	89.68	62.78
NuMZn3-AF	21.87	61.24	76.55	87.49	61.24
NuMZn1-TL	20.23	56.65	70.81	80.92	56.65
NuMZn2-TL	20.28	56.78	70.97	81.11	56.78
NuMZn3-TL	20.72	58.01	72.52	82.88	58.01
NuQMZn1-AF	28.23	79.04	98.80	112.91	79.04
NuQMZn2-AF	26.73	74.85	93.56	106.92	74.85
NuQMZn3-AF	27.63	77.37	96.71	110.53	77.37
NuQMZn1-TL	22.88	64.06	80.08	91.51	64.06
NuQMZn2-TL	21.65	60.63	75.79	86.61	60.63
NuQMZn3-TL	21.97	61.52	76.90	87.88	61.52

^a^ Percentage of Estimated Average Requirement (EAR) for zinc supplied by consumption of 0.5 ears per pre-school child (1–3 years old), 1 ear per child (4–8 years old) per day and 2 ears per child (9–13 years old) or pregnant women per day, assuming an average ear weight of 140 g (DW). The amount per day recommended is 2.5, 4, 7 and 10 mg for pre-school children, 4–8 years old children, 9–13 years old children and pregnant women, respectively (Values from the DRI reports). Source: National Institutes of Health (NIH) https://ods.od.nih.gov/factsheets/Zinc-HealthProfessional/, accessed on 7 December 2021.

## Data Availability

Data is contained within the article.

## Appendix A

**Table A1:** Percentage of Estimated Average Requirement (EAR) for iron supplied via the consumption of 0.5 ears per pre-school child (1–3 years old), 1 ear per child (4–8 years old) per day and 2 ears per child (9–13 years old) or pregnant women per day, assuming an average ear weight of 140 g (DW).

Genotype	Iron in Fresh Maize (mg kg^−1^ DW)	Pre-School Children (1–3 Years Old)	Children (4–8 Years Old)	Children (9–13 Years Old)	Pregnant Women (19–50 Years Old)
% EAR					
Normal1-AF	12.77	29.81	43.62	60.62	15.55
Normal2-AF	13.01	30.36	44.42	61.74	15.84
Normal3-AF	13.52	31.54	46.16	64.16	16.46
NuMZn1-TL	11.87	27.69	40.52	56.32	14.45
NuMZn2-TL	11.37	26.54	38.84	53.98	13.85
NuMZn3-TL	11.92	27.82	40.70	56.57	14.51
NuMZn1-AF	14.26	33.28	48.71	67.69	17.36
NuMZn2-AF	13.94	32.53	47.60	66.15	16.97
NuMZn3-AF	13.92	32.48	47.54	66.07	16.95
Normal1-TL	14.14	33.00	48.29	67.12	17.22
Normal2-TL	13.76	32.10	46.98	65.29	16.75
Normal3-TL	13.92	32.47	47.52	66.04	16.94
NuQMZn1-AF	13.17	30.73	44.97	62.51	16.03
NuQMZn2-AF	12.61	29.43	43.07	59.86	15.36
NuQMZn3-AF	12.72	29.68	43.44	60.37	15.49
NuQMZn1-TL	12.40	28.93	42.33	58.84	15.09
NuQMZn2-TL	12.88	30.06	43.99	61.13	15.68
NuQMZn3-TL	12.12	28.27	41.37	57.50	14.75

The amount per day recommended is 3, 4.1, 5.9, and 23 mg for pre-school children, 4–8 years old children, 9–13 years old children and pregnant women, respectively (values from the DRI reports). Source: National Institutes of Health (NIH) https://ods.od.nih.gov/factsheets/Iron-HealthProfessional/, accessed on 5 April 2022.
